# Comparison of the Structure, Mechanical Properties and Effect of Heat Treatment on Alloy Inconel 718 Produced by Conventional Technology and by Additive Layer Manufacturing

**DOI:** 10.3390/ma16155382

**Published:** 2023-07-31

**Authors:** Martin Švec, Pavel Solfronk, Iva Nováková, Jiří Sobotka, Jaromír Moravec

**Affiliations:** Department of Engineering Technology, Faculty of Mechanical Engineering, Technical University of Liberec, Studentská 1402/2, 461 17 Liberec, Czech Republic

**Keywords:** nickel-based alloy Inconel 718, selective laser melting, high-temperature mechanical properties, structure, impact energy, hardness

## Abstract

The nickel-iron-based alloy Inconel 718 is a progressive material with very good mechanical properties at elevated and lower temperatures. It is used both as wrought and cast alloys as well as material for additive manufacturing technologies. This is the reason why it has received so much attention, as supported by numerous publications. However, these are almost exclusively focused on a specific type of production and processing, and thus only report differences in the mechanical properties between samples prepared by different technologies. Therefore, the major aim of this research was to show how the structure and mechanical properties differ between samples produced by conventional production (wrought alloy) and additively manufactured SLM (Selective Laser Melting). It is shown that by applying appropriate heat treatment, similar strength properties at room and elevated temperatures can be achieved for SLM samples as for wrought samples. In addition, the mechanical properties are also tested up to a temperature of 900 °C, in contrast to the results published so far. Furthermore, it is proven that the microstructures of the wrought (here rolled) and SLM alloys differ significantly both in terms of grain shape and the size and distribution of precipitates.

## 1. Introduction

Inconel 718 is a precipitation-hardenable nickel-iron-based alloy, which also contains chromium, niobium, molybdenum and other elements, which exhibits high strength at room and elevated temperatures, good corrosion resistance, high fatigue life and high creep strength. Due to its high nickel content, Inconel 718 also exhibits good properties at low temperatures [1,2]. At the same time, it is characterized by good weldability. However, when welding Inconel 718, the welding conditions have to be precisely monitored because this alloy tends to form micro-cracks in the heat-affected zone (HAZ) due to the precipitation of carbide particles along the grain boundaries [3].

For these reasons, Inconel 718 is a suitable candidate to be used in high-temperature applications up to 700 °C. It is currently used in a wide range of engineering applications, e.g., in the manufacturing of gas turbine parts, turbocharger rotors, steam turbine flow-through parts or rotating parts of aircraft engines [4,5,6]. Nevertheless, the high hardness and low thermal conductivity of this material is a problem in the processing of this alloy. As a result, Inconel 718 is difficult to be machined by a conventional machining device [1]. These two factors, together with the fact that the parts produced from Inconel 718 are mostly complex in shape concerning the field of application, have resulted in the increased use of additive manufacturing (e.g., SLM) for the processing of Inconel 718. The SLM manufacturing process allows (among others) a significant reduction in the technological production steps and a high percentage of material utilization [7], which is one of the main potential benefits of additive manufacturing technology for the production of products from Inconel 718, considering the high price of nickel.

The microstructure of alloy Inconel 718 consists of a γ matrix (Ni, Cr) with an FCC (face-centred cubic) lattice, which is hardened by a substitutional solid solution. The solid solution alone would not ensure its refractoriness. Therefore, Inconel 718 is alloyed with other elements (e.g., aluminium, titanium, niobium, chromium and so on) to form hardening carbide or intermetallic phases. The most common hardening phases observed in Inconel 718 ductile alloy include [1,8,9,10,11]:

1. γ′—Ni_3_(Al, Ti, Nb) with face-centred L12 lattice
2. γ″—Ni_3_Nb with body-centred D022 lattice
3. δ—Ni_3_(Nb, Ti) with orthorhombic D0a lattice
4. Carbides (Nb, Ti)C with face-centred B1 lattice
5. Laves phases (Ni, Fe, Cr)_2_(Nb, Mo, Ti) with hexagonal C14 lattice

The γ′ and γ″ phases, which are coherent with the γ matrix, have the largest hardening effect. By using a special hardening heat treatment, a quantity of 4% of the γ′ phase and 16% of the γ″ phase can be obtained, resulting in a maximum hardening effect [1,12]. A study [2] has proven that when the alloy contains up to 3.5% titanium and up to 6% niobium, both the γ′ and γ″ phases are formed in the structure. If the titanium content exceeds 3.5%, only the γ′ phase precipitates. Conversely, if the alloy contains more than 6% niobium, only the γ″ phase is formed. As it has been shown, e.g., in [13], in the basic delivery state, precipitates of the δ phase occur at grain boundaries in Inconel 718 alloy; they are formed during forging and pin up the grain boundaries. This results in a regular fine-grain structure [13,14]. The formation of γ′ and γ″ is only achieved after hardening heat treatment. If the Inconel 718 alloy is exposed to high temperatures for a long time or if overaging occurs during the heat treatment, the coherent γ′ and γ″ phases are converted back to incoherent δ precipitates, which results in a simultaneous decrease in strength [15]. In terms of grain character, Inconel 718 produced by casting and forming is typically characterized by fine equiaxed grains with a large proportion of twins [16].

When Inconel 718 is produced by SLM, high residual stresses are generated in the structure, which can result in the transformation of the precipitates present into the δ phase, which is preferentially distributed at grain boundaries [17]. Some studies, e.g., [18], have shown that larger amounts of the δ phase can cause dislocation piling up in the process of tensile testing at elevated temperatures, leading to local stress concentration and formation of microcracks in the material. On the other hand, the lack of a δ phase reduces the strength of grain boundaries at high temperatures. The amount of the δ phase (and so also modification of the mechanical properties) can be controlled by a suitably chosen heat treatment [13,18]. The grains of material produced by SLM technology reveal a columnar structure, due to the rapid solidification of thin layers and unidirectional heat dissipation [7,16]. The aforementioned differences in the microstructures between the SLM and rolled samples result in lower plasticity and inferior creep properties of the material produced by SLM technology. Creep lifetime can be reduced up to three times in the case of using SLM fabrication technology [16]. Nevertheless, experiments have been carried out with lattice structures, which are topologically ordered (3D open-celled structures), composed of one or more repeating unit cells. These lattice structures manufactured by SLM technology exhibited high specific strength and were characterized by the ability to widely modify their deformation behaviour. Such SLM lattice structures offer a wide range of potential applications—not only in the field of mechanical products but also, e.g., in biomedicine [19].

Another challenge of SLM production is the need for very precise testing and adjustment of the process parameters for a specific alloy type. The appropriate process parameters significantly affect the final properties of the produced material. If the parameters are chosen incorrectly, excessive thermal internal stresses or an unacceptable level of local voids (the portion of pores must be lower than 0.5%) are created in the part, resulting in a decrease in mechanical properties for the given manufactured material [7,20,21].

In the case of production alloy Inconel 718 (whether cast, wrought or produced by SLM technology), it is necessary to carry out subsequent heat treatment with precise control of the process parameters. A suitably selected heat treatment regime leads to a decrease in the residual stresses; a reduction in the amount of certain phases, which can degrade the mechanical properties; and last but not least, the precipitation of the hardening γ′/γ″ phases that have a strong hardening effect on the alloy. The heat treatment of alloy Inconel 718 is generally carried out in two steps. First, a solution annealing is carried out, which dissolves the δ phase and other present phases (e.g., the Laves phase) into the matrix. Solution annealing is performed at temperatures above 1000 °C, as this is the limiting temperature for the stability of the δ and Laves phases. The dwell time of the solution annealing is short (1 h), so that no grain coarsening occurs, which is no longer fixed by δ phase precipitates at the grain boundaries. This is followed by the hardening heat treatment, which is carried out at lower temperatures and for longer times [13,22,23]. After heat treatment, the alloy shows high mechanical properties, but its ductility and notch toughness decrease significantly [17,24,25,26].

The present study deals with the comparison of the microstructural and mechanical properties of alloy Inconel 718 produced both by standard technologies (casting and forming) and by SLM technology. In addition, the effect of heat treatment on both types of samples (denoted as rolled and SLM samples) is also studied.

Because of the above, the research was focused on the possibility of applying heat treatment to samples prepared by the SLM method. As a result, similar strength properties to those of the rolled specimens were obtained. At the same time, the effect of heat treatment on the rolled and SLM samples was compared both in terms of mechanical properties and microstructure (grain shape and size and precipitate size and distribution).

For application utilization, knowledge of the properties is important not only in terms of the thermal availability of the materials but also at even higher temperatures. From this, further behaviour of the product can be deduced. However, the vast majority of papers focus on the assessment of the Inconel 718 alloy properties up to 700 °C, and, in exceptional cases up to 800 °C. In this study, the properties of rolled and SLM specimens were tested up to 900 °C.

At high temperatures (900 °C and above), Inconel 718 reveals superplastic properties [27,28]. It has been described in the literature [27] that in the temperature interval 900–1000 °C, this alloy does not show signs of dynamic recrystallization. Tests at 900 and 950 °C proved that the only active recovery mechanism is the dynamic recovery. At the same time, however, particle precipitation occurs to compensate for such recovery from the recrystallization. Deformation mechanisms are controlled by the glide and climb of dislocations, as long as precipitation does not occur. Deformation is basically governed by the self-diffusion of Ni [27].

## 2. Materials and Methods

Within the framework of this study, the material Inconel 718 produced by the classical method (casting and subsequent rolling), as well as the material produced by additive technology using the SLM (Sintering Laser Melting) method, were experimentally tested and investigated. The chemical composition of the rolled Inconel 718 was determined using a Q4 TASMAN optical spectrometer (Bruker Elemental GmbH, Germany) and is generally summarized in Table 1.

**Figure 1:**
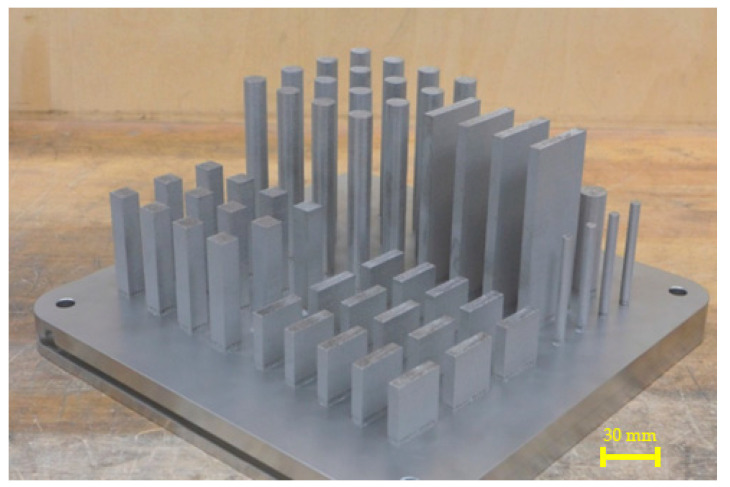
Detail of the fabricated samples with support structures.

Three-dimensionally-printed samples were produced on a SLM280HL machine (SLM Solution AG, Lübeck, Germany) equipped with a Yb:YAG laser unit. The powder for 3D printing was supplied by the SLM Solutions Group AG. The particle size of the powder varied from 13 to 88 µm, appropriate for use with additive SLM technology. The chemical composition of the powder is summarized in Table 2. The definition of the samples for 3D printing was carried out in Materialise Magics 23.1 (Materialise NV, Leuven, Belgium). To minimize internal stresses, the samples were positioned as to occupy the smallest possible ground area and to have a minimum amount of support structures. The actual samples were set up on the printing surface at a distance of 3 mm above the build platform. Connection of the samples to the platform was accomplished with block support structures. The selected process paramaters resulted from the process optimalization. Fabricated samples are shown in Figure 1 and process parameters used for their fabrication are shown in Table 3.

The microstructures of the samples were studied on metallographic scratch patterns using a Tescan Mira 3 electron microscope (Tescan Orsay Holding a.s., Brno, Czech Republic) equipped with an Oxford UltimMax65 energy-dispersive detector (Oxford Instruments plc, Oxfordshire, England) for local chemical analysis. The samples were prepared by the standard metallographic method (grinding, polishing and final polishing step with OP-S suspension). Electrolytic polishing was not applied. Grain size and orientation were analysed with an Oxford SYMMETRY EBSD detector (Oxford Instruments plc, Oxfordshire, England). The phase compositions of alloys were verified by X-ray diffraction (XRD) using an X’Pert^3^ Powder diffractometer (PANalytical, Almelo, Netherlands) in Bragg-Brentano geometry (Co Kα radiation, λ = 1.78901 Å).

**Figure 2:**
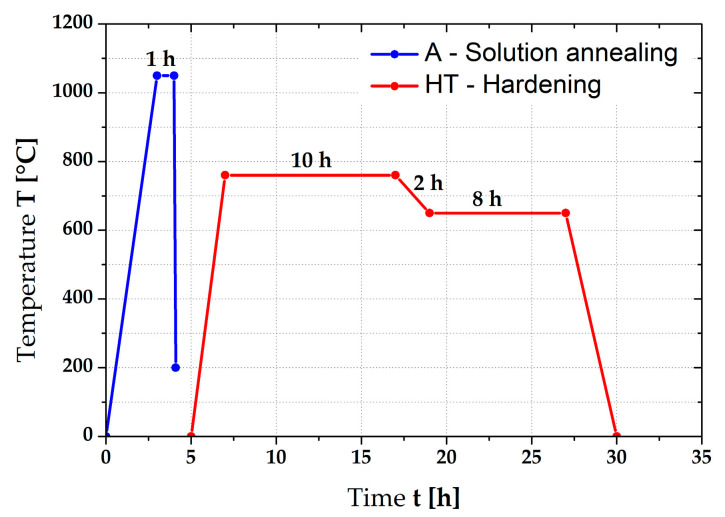
Graphical illustration of the alloy Inconel 718 heat treatment.

To study the effect of heat treatment on the structure and mechanical properties of Inconel 718, solution annealing (marked as “A”) and then a precipitation hardening (marked as “HT”) were carried out. During heat treatment of Inconel 718, it is truly crucial to precisely observe the temperatures and dwell time to achieve the optimum mechanical properties [22]. The heat treatment was carried out under the following conditions (see Figure 2):

1. Solution annealing at temperature 1050 °C for 1 h, rapid cooling in water (marked as “A”).
2. Precipitation hardening at temperature 760 °C for 10 h (heating rate 5 °C/min), subsequent cooling from 760 to 650 °C at cooling rate 0.92 °C/min and dwell time at temperature 650 °C for 8 h, air cooling.

The heat treatment was carried out in furnace 11016S Classic using a shielding atmosphere of argon. The selected temperature cycle was chosen on the basis of [22] and the results of the hardness and microstructure evaluation of the prepared samples.

The mechanical properties of the tested material in the basic state and after heat treatment were tested at room and elevated temperatures (600, 700, 800 and 900 °C) on a Zwick Kappa machine 50 SS-CF (ZwickRoell AG, Ulm, Germany). The samples were prepared according to [29] (diameter 8 mm and length 95 mm) by turning. The dwell time at each tested temperature before the starting of measurement of the mechanical properties was 10 min. The heating of samples during the test was carried out in a Zwick Roell MPFU-10 temperature chamber. The accuracy of this temperature chamber was ±1 °C. The strain rate was 0.00025 s^−1^. The initial length of the measured section was chosen to be 40 mm. The values of the mechanical properties were average values from the measurements of 5 samples under the same conditions. The measured mechanical values differed insignificantly; therefore, the standard deviation was not plotted in the relevant figures to keep their clarity.

The hardness of the samples was measured on the surface of the metallographic samples by the Vickers method at a load of 10 kp (HV10) according to standard ČSN EN ISO 6507-1 on a Qness Q30A device (ATM Qness GmbH, Mammelzen, Germany).

The notch toughness was tested by the Charpy method on a LabTest CHK 450 J- I machine (Labortech s.r.o., Opava, Czech Republic). The testing samples had dimensions of 10 × 10 × 55 mm and they were V-notched.

Marking of the samples for determination the mechanical properties and for microscopic evaluation was chosen as follows:

- Rolled material without heat treatment—ACR;
- Rolled material with heat treatment “HT” in accordance with Figure 2—ACR HT;
- Rolled material after the solution annealing (1050 °C; 1 h)—ACR A;
- Rolled material without heat treatment after the high-temperature tensile tests—ACR TT600, ACR TT700, ACR TT800, ACR TT900;
- Rolled material with heat treatment “HT” after the high-temperature tensile test—ACR HT TT600, ACR HT TT700, ACR HT TT800, ACR HT TT900;
- Three-dimensionally-printed material without heat treatment—SLM;
- Three-dimensionally-printed material with heat treatment “HT” in accordance with Figure 2—SLM HT;
- Three-dimensionally-printed material without heat treatment after the high-temperature tensile test—SLM T600, SLM TT700, SLM TT800, SLM TT900;
- Three-dimensionally-printed material with heat treatment “HT” after the high-temperature tensile test—SLM HT TT600, SLM HT TT700, SLM HT TT800, SLM HT TT900.

## 3. Results

### 3.1. The Structure of the Investigated Alloys

**Figure 3:**
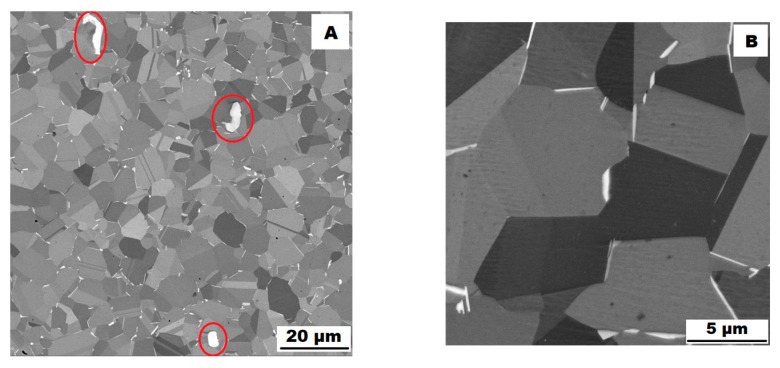
Microstructure of alloy ACR (SEM, 10 kV, BSE): (**A**) Overview of the structure—coarse (Nb, Ti)C carbide precipitates are highlighted by the red circles, all other particles are δ phases; (**B**) Detail of the structure with particles of δ phase.

**Figure 4:**
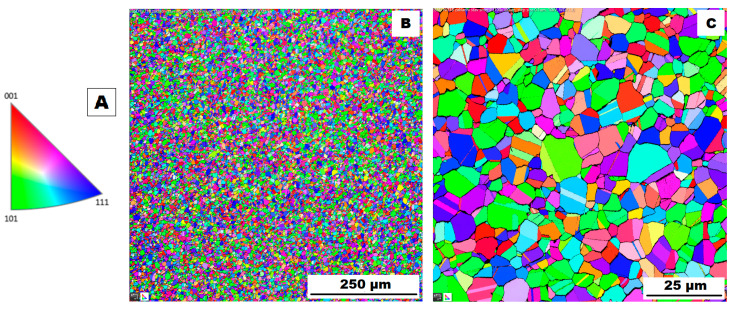
EBSD map of alloy ACR: (**A**) The orientation map; (**B**) SEM, 10 kV, area 700 × 700 µm, step size 0.7 µm; (**C**) SEM, 10 kV, area 100 × 100 µm, step size 0.1 µm.

**Figure 5:**
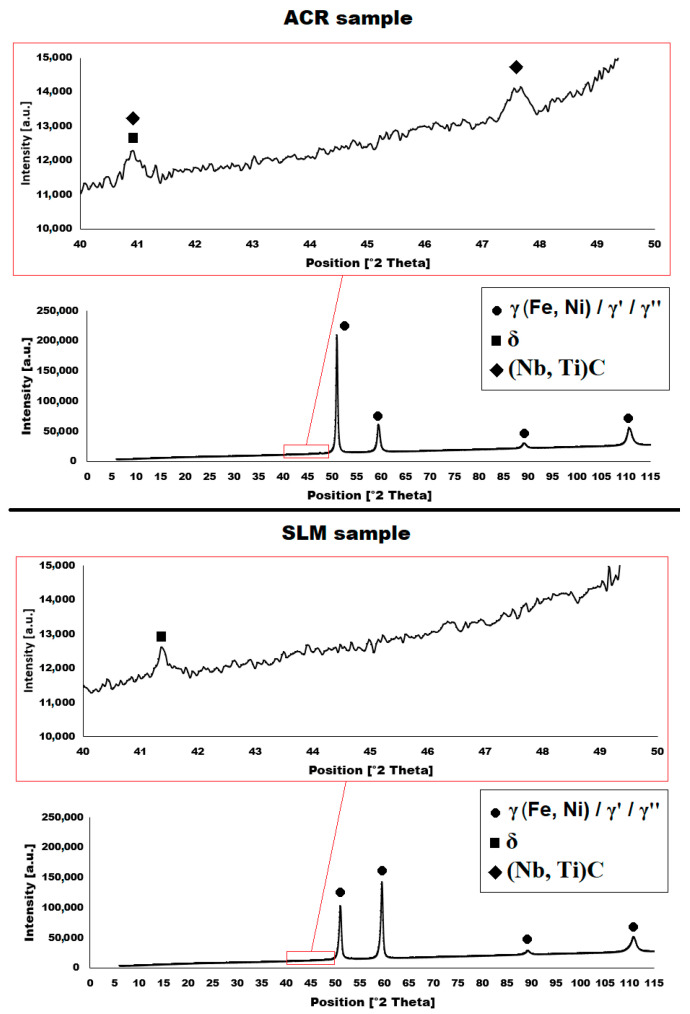
XRD diffractograms of alloys ACR and SLM.

**Figure 6:**
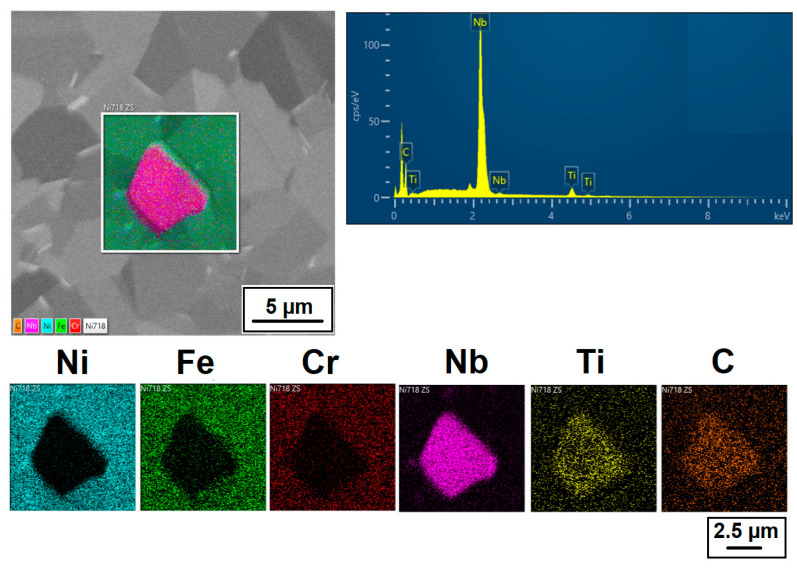
EDX analysis of the (Nb, Ti)C carbide precipitates in material ACR (SEM, 10 kV).

The structure of the ACR alloy is shown in Figure 3A,B. The microstructure was determined in two mutually perpendicular directions (in the rolling direction and the direction perpendicular to the rolling direction), but no significant differences were observed between these directions. From Figure 3A and the EBSD map in Figure 4B,C, it is obvious that the structure was composed of equiaxed grains whose average size was determined to be 9.18 ± 1.20 µm based on EBSD measurements. Figure 4A shows an orientation map, which is the same for all used EBSD analyses. Numerous twins were observed in the grain structure (Figure 4C) as well. Small needle-shaped δ phase precipitates with thicknesses on the order of hundreds of nanometres and lengths on the order of units of micrometres were found at the grain boundaries (see Figure 3B and XRD in Figure 5). Considering the peak overlaps in the diffractogram (similarly to e.g., [8]), the presence of the δ phase was confirmed by the chemical composition of the precipitates measured by the EDX method, by which the high nickel and niobium content in the particles was measured. Moreover, coarse precipitates of about 10 µm were observed in the structure (highlighted in Figure 3A). These secondary particles were identified as (Nb,Ti)C-type carbides by XRD (see Figure 5) and by EDX analysis (see Figure 6).

Also, the literature [30,31] confirms that an MC carbide phase is formed in Inconel 718, whose lattice parameter lies between those reported for NbC and TiC, being closer to the lattice parameter for NbC. Therefore, it can be concluded that complex carbides are formed in Inconel 718, in which part of the niobium is substituted by titanium.

**Figure 7:**
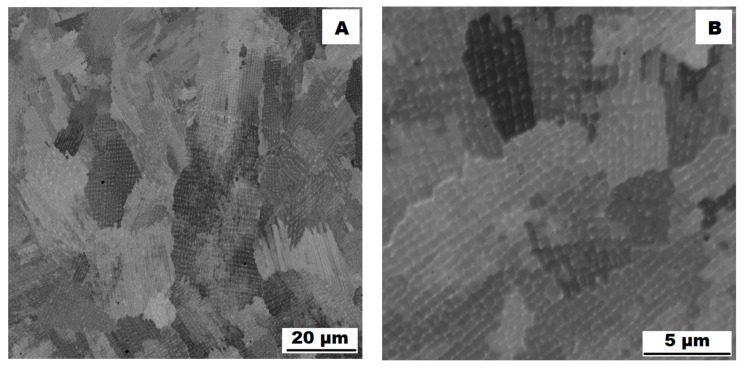
Microstructure of alloy SLM (SEM, 10 kV, BSE): (**A**) Overview of the structure; (**B**) Detail of the structure.

**Figure 8:**
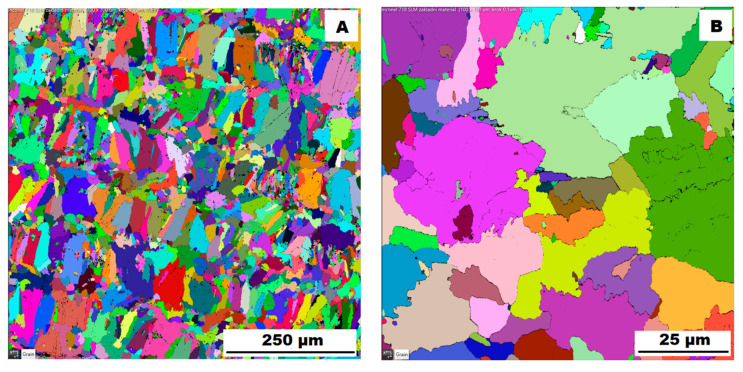
EBSD map of alloy SLM: (**A**) SEM, 10 kV, area 700 × 700 µm, step size 0.7 µm; (**B**) SEM, 10 kV, area 100 × 100 µm, step size 0.1 µm.

The microstructure of the material produced by the additive technology (SLM samples) was quite different from that of the ACR. The structure of the SLM samples is shown in Figure 7A,B. In the case of 3D-printed samples, the grains had an irregular, sometimes “dendritic” shape, with a strong columnar elongation in one of the directions (see Figure 7A and the EBSD map in Figure 8A). The grain sizes were larger and showed a significantly larger standard deviation (17.51 ± 10.99 µm) compared with the rolled samples. Secondary particles were present as a dense network of very fine δ phase precipitates with dimensions on the order of tens to hundreds of nanometres (see detail of the structure in Figure 7B and XRD in Figure 5. Coarse carbide secondary particles were not observed in this structure—see the absence of the (Nb, Ti)C peak in the SLM diffractogram in Figure 5. Also, twinning was not observed in the 3D-printed material (see Figure 8B).

### 3.2. Effect of Heat Treatment on the Structure of the Tested Alloys

**Figure 9:**
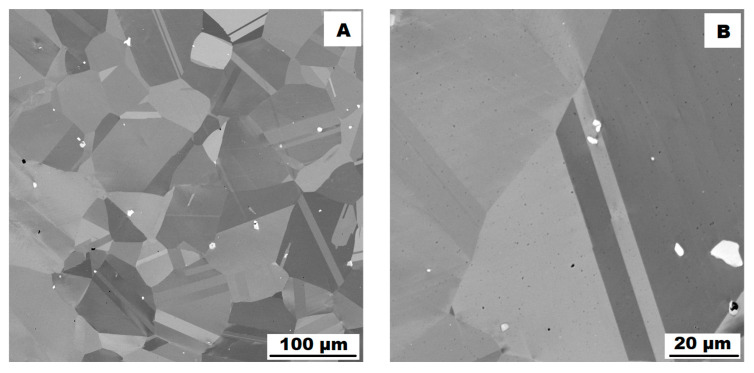
Microstructure of alloy ACR HT (SEM, 10 kV, BSE): (**A**) Overview of the structure; (**B**) Detail of the structure.

**Figure 10:**
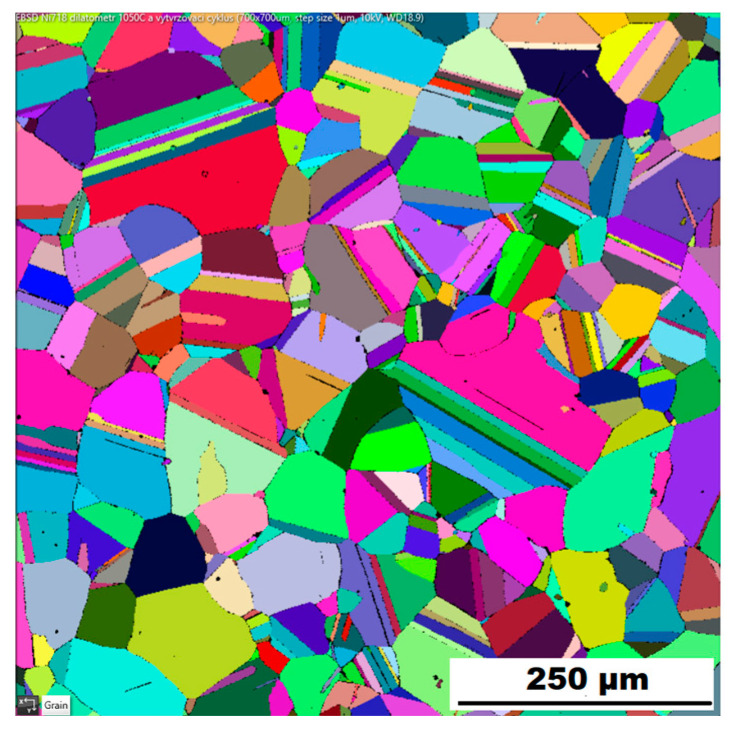
EBSD map of alloy ACR HT (SEM, 10 kV, area 700 × 700 µm, step size 0.7 µm).

Application of the solution annealing and hardening (heat treatment “HT”) to the ACR samples resulted in significant coarsening of the matrix grains—see the images of the structure in Figure 9A,B and the EBSD map in Figure 10 (ACR HT samples). The grain size was determined to be 32.65 ± 20.28 µm by the EBSD analysis. The high value of the standard deviation indicates increased inhomogeneity in grain size after application of the “HT” heat treatment. A large number of twins were again observed in the structure of the ACR HT material.

The heat treatment also affected the phase composition. Coarse carbide particles of (Nb, Ti)C type remained in the structure. Their sizes were around units to tens of micrometres—see Figure 9A,B. However, the tiny needle-shape δ phase precipitates that were presented at grain boundaries in the ACR state dissolved into the matrix during the “HT” heat treatment and were replaced by coherent γ′ and γ″ precipitates [3,13].

**Figure 11:**
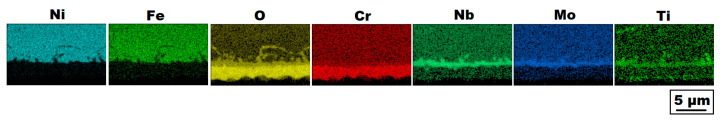
EDX analysis of the material ACR HT surface without the use of shielding atmosphere—there is evident creation of oxide layers on the surface (SEM, 10 kV).

Heat treatment of the Inconel 718 must be performed in a shielding atmosphere; otherwise, a double layer of chromium oxides and mixed oxides of niobium, titanium and molybdenum will be formed on the surface of a material (see EDX maps in Figure 11). These oxide layers passivate the surface of a sample, thus preventing the diffusion of elements from the surface. Among other effects, this prevents the application of a chemical heat treatment, if required, within the tribological requirements.

**Figure 12:**
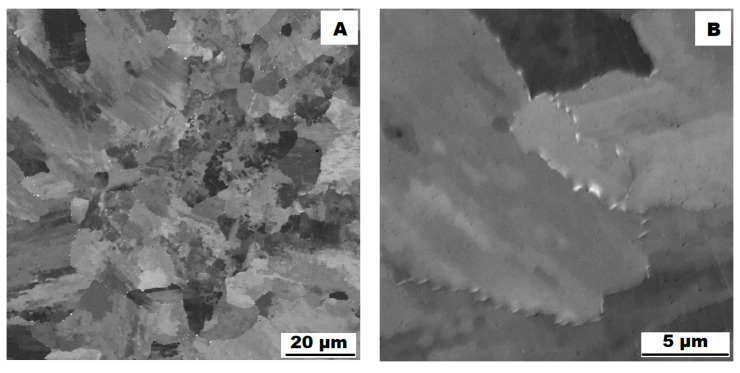
Microstructure of alloy SLM HT (SEM, 10 kV, BSE): (**A**) Overview of the structure; (**B**) Detail of the structure.

The structure of the 3D-printed material after “HT” heat treatment (SLM HT samples) is shown in Figure 12A,B. The distribution of the secondary particles was different compared with the SLM condition. While the SLM material formed a network of small precipitates (Figure 7B), the “HT” heat treatment caused the dissolution of this network and the δ phase precipitates remained only at the grain boundaries in the form of fine spheres or needles. Heat treatment further caused the formation of strongly hardening coherent γ′ and γ″ phases.

**Figure 13:**
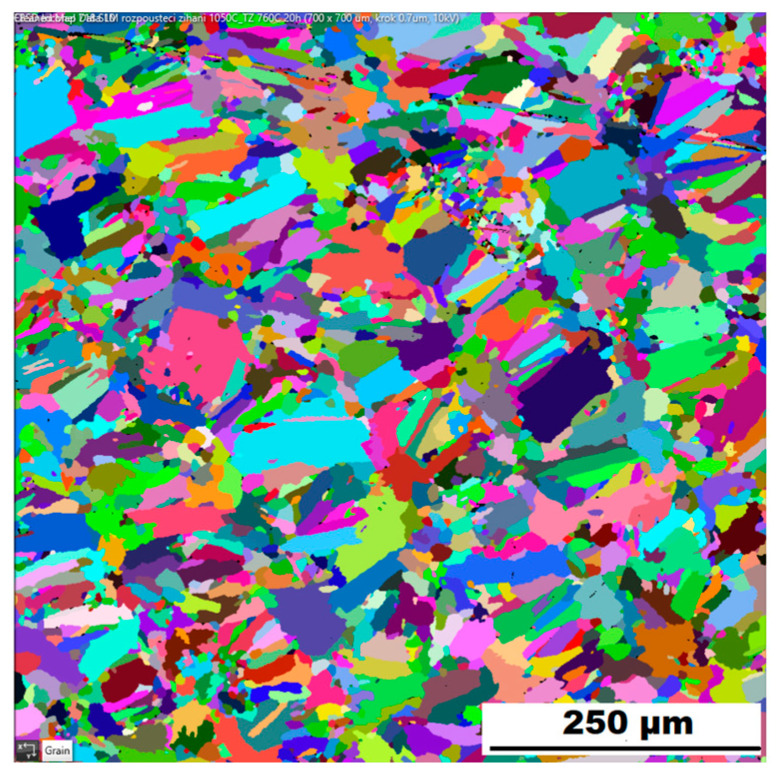
EBSD map of alloy SLM HT (SEM, 10 kV, area 700 × 700 µm, step size 0.7 µm).

Heat treatment of the “HT” 3D-printed samples revealed a minimal effect on the grain character and size (see the EBSD map in Figure 13). The mean grain size was 17.26 ± 10.90 µm. Again, the values of the standard deviation after “HT” remained similar to those of the samples produced by the SLM method. Table 4 gives an overview of grain size. From Table 4 is clear that the ACR specimen revealed significant grain refinement by rolling. Heat treatment (ACR HT) resulted in rapid grain coarsening after exceeding 1000 °C. This is due to the dissolution of the σ phase, which accelerated the growth of the grains by decreasing the resistance of the migration of the grain boundary [28]. This phenomenon was not observed in the case of SLM samples, where grain sizes before and after heat treatment remained unchanged. This is probably due to the fact that the basic state of the 3D-printed samples (SLM samples) is casted; therefore, temperatures exceeded 1000 °C during the production.

### 3.3. Mechanical Properties at Room and High Temperatures

The measured yield strengths at room temperature for all states of rolled and 3D-printed materials are compared in Table 5. The ACR samples have a yield stress of 540 MPa. The SLM samples revealed a slightly higher yield strengthof 581 MPa. Solution annealing “A” caused the yield strength to decrease up to 304 MPa (ACR sample A). The hardening heat treatment “HT” led to a significant increase in yield strength at room temperature up to 1147 MPa (ACR HT sample). A similar value was also measured for the SLM HT samples—in this case, 1190 MPa.

**Figure 14:**
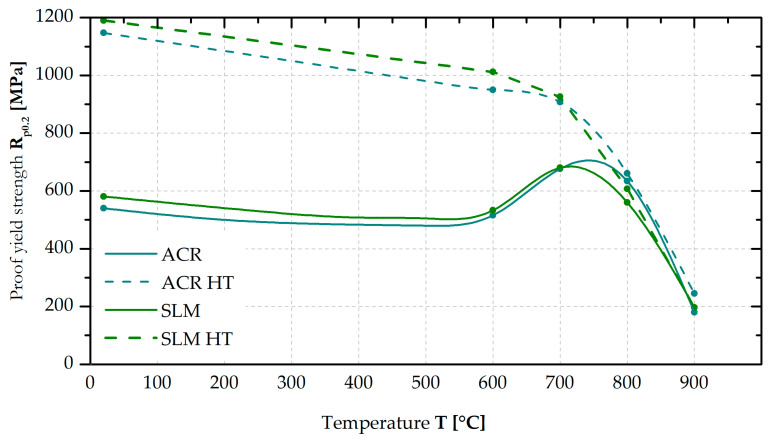
Proof yield strength vs. temperature (in the state without and with heat treatment “HT”).

The yield strength (YS) values at 600, 700, 800 and 900 °C were measured for the ACR, ACR HT, SLM and SLM HT samples (see Figure 14). From the graph, it can be seen that the trend of the yield strength values for ACR and SLM samples does not differ significantly over the entire monitored interval. Only at 800 °C was the yield strength value for ACR samples measured to be approximately 70 MPa higher than that of the SLM samples. At 700 °C, both tested material types showed yield strength anomalies. Both the ACR HT and SLM HT samples show a similar pattern of yield strength values over the entire monitored temperature range, regardless of their manufacturing technology. A slightly higher deviation in the yield strength value was determined at 600 °C, where SLM HT samples had a 70 MPa higher yield strength value, and at 800 °C, where, on the contrary, ACR HT samples had yield strength value about 55 MPa higher. A positive finding is that both the ACR HT and SLM HT samples still revealed yield stress exceeding 600 MPa at 800 °C.

**Figure 15:**
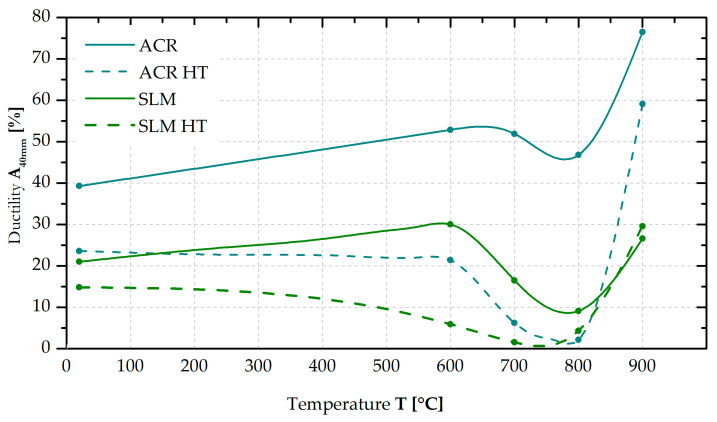
Ductility vs. temperature (in the state without and with heat treatment “HT”).

Quite a problematic aspect of using alloy Inconel 718 rests in its highly temperature-dependent ductility (see Figure 15). While the ACR material exhibits a ductility of A_40mm_ = 39% at room temperature, its ductility increases with increasing temperature up to A_40mm_ = 77% at 900 °C. In the temperature region around 700 and especially 800 °C, it was shown that the trend of the material ductility had been changed (ductility between 600 and 800 °C decreased), which may be related to the yield strength anomaly.

For the SLM samples, a very similar trend of ductility versus temperature was observed in the case of the ACR samples (see Figure 15). However, the ductility values at all temperatures were twofold (at 20 and 600 °C), threefold (at 700 and 900 °C) and fivefold (at 800 °C) lower for the SLM samples.

After the “HT” heat treatment, there is a significant reduction in ductility values for both the formed and 3D-printed samples over the entire temperature range. Such a phenomenon is related to the hardening effect of testing samples. While the ACR HT samples show a more significant decrease in ductility after 600 °C, the SLM HT samples reveal a gradual decrease in ductility with increasing temperature. In any case, the ductility of the ACR HT samples in the temperature interval 700–800 °C and of the SLM HT samples in the temperature interval 600–800 °C did not exceed 6%.

**Figure 16:**
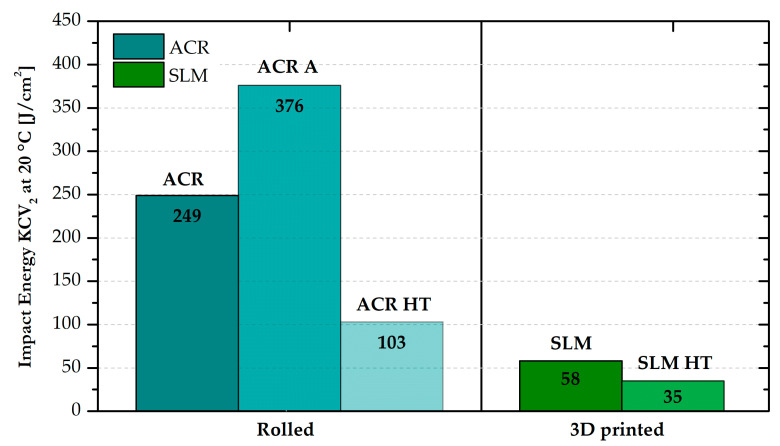
Values of impact energy at room temperature for all states of formed and 3D-printed samples of alloy Inconel 718.

As part of the study of Inconel 718, the notch toughness at room temperature was also measured. The resulting values for all states of the rolled and 3D-printed alloys are graphically shown in Figure 16. For the ACR material, an impact energy value of 249 J·cm^−2^ was measured. After solution annealing “A”, there was observed a strong increase up to 376 J·cm^−2^. Then, after hardening of the material (ACR HT), there was a significant decrease in the impact energy value to 103 J·cm^−2^. The 3D-printed samples showed significantly lower impact energy values compared with the formed samples both in the SLM condition (58 J·cm^−2^) and after heat treatment “HT”—SLM HT (35 J·cm^−2^).

**Figure 17:**
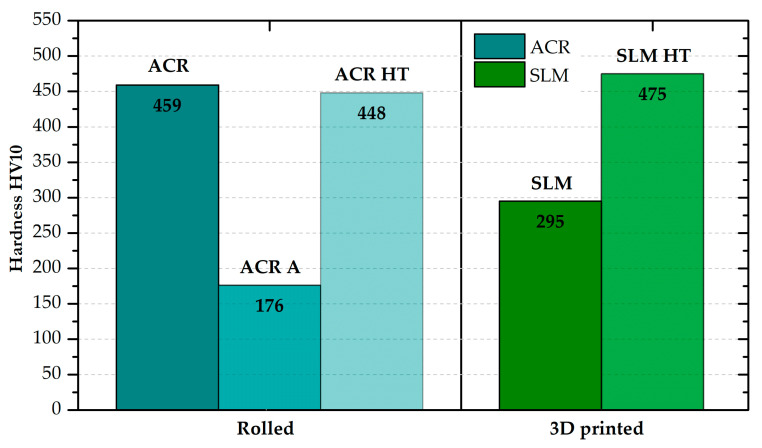
Values of hardness for all states of formed and 3D-printed samples of alloy Inconel 718.

A difference in material hardness was also observed between the rolled and 3D-printed samples (see Figure 17). However, this difference was only determined for the materials in the basic state. After “HT” heat treatment, the hardness values for the rolled and 3D-printed material were comparable. From the comparison of the ACR and ACR HT samples, it can be seen that the measured hardness values are almost identical. However, the yield strength values are significantly different (540 MPa—ACR vs. 1147 MPa—ACR HT).

For the rolled material, the hardness was also measured after solution annealing “A”, where the hardness expectedly decreased to 178 HV10.

## 4. Discussion

From the comparison of structures and yield strengths at 20 °C (see Table 5) for ACR samples (Figure 3A,B) and for ACR HT samples (see Figure 9A,B), it is evident that performed heat treatment had two major effects on the alloy:

1. There was grain coarsening after the heat treatment.
2. During the heat treatment (samples ACR HT), the needle-shape δ phase precipitates were dissolved into the matrix and replaced by strongly hardening coherent γ′ and γ″ phases. Thus the resulting alloy hardening is given by a combination of solid solution hardening and precipitation hardening—(Nb, Ti)C + γ′/γ″ for the ACR HT samples and δ + (Nb, Ti)C for the ACR samples.

A comparison of the measured high-temperature yield strengths for the individual samples (see Figure 14) shows that the samples without heat treatment (ACR and SLM material) exhibit significantly lower yield strength up to 700 °C than the corresponding samples with the heat treatment of material (ACR HT and SLM HT). This is probably due to the formation of strongly hardening γ′/γ″ phases in the structure of the heat-treated samples (HT).

**Figure 18:**
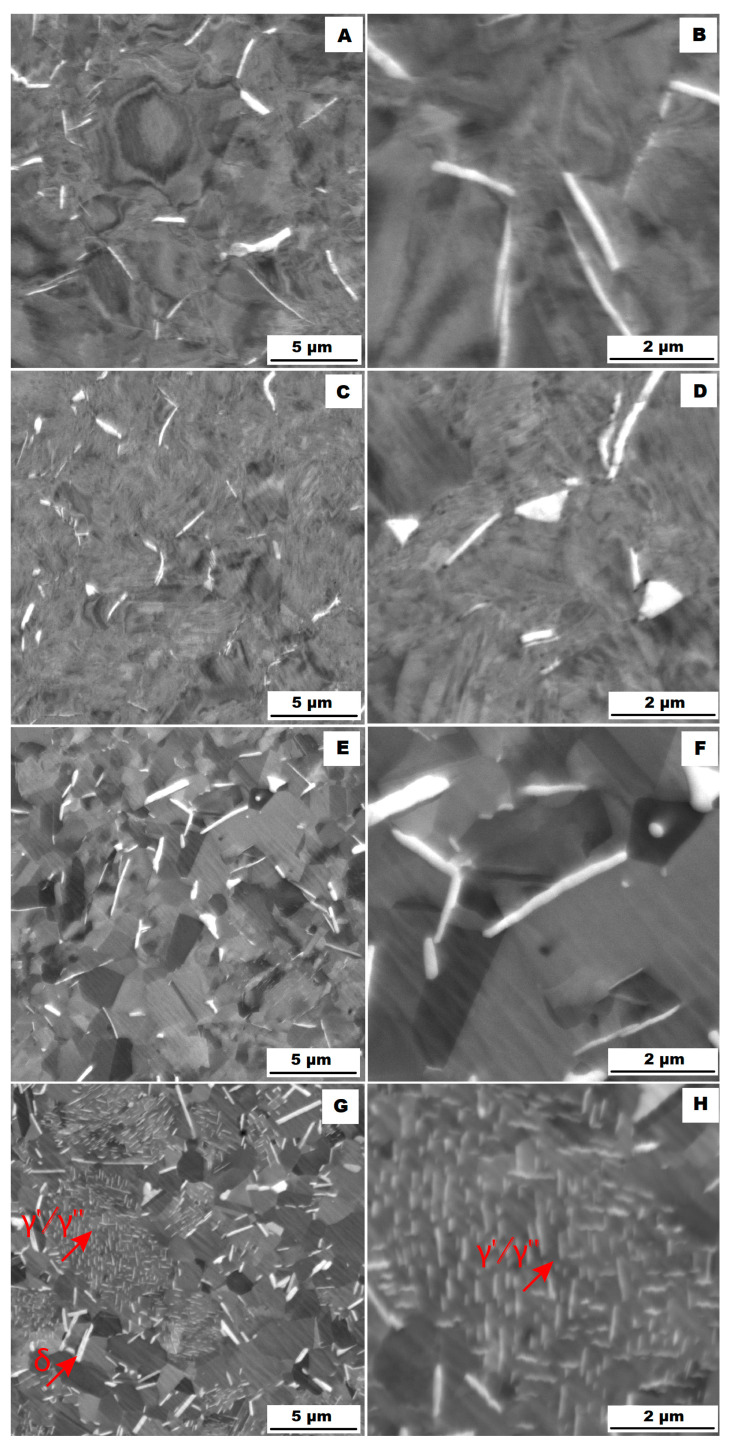
Microstructures of samples ACR after the tensile tests (SEM, 10 kV, BSE): (**A**,**B**) tensile test at 600 °C (ACR TT600); (**C**,**D**) tensile test at 700 °C (ACR TT700); (**E**,**F**) tensile test at 800 °C (ACR TT800); (**G**,**H**) tensile test at 900 °C (ACR TT900).

**Figure 19:**
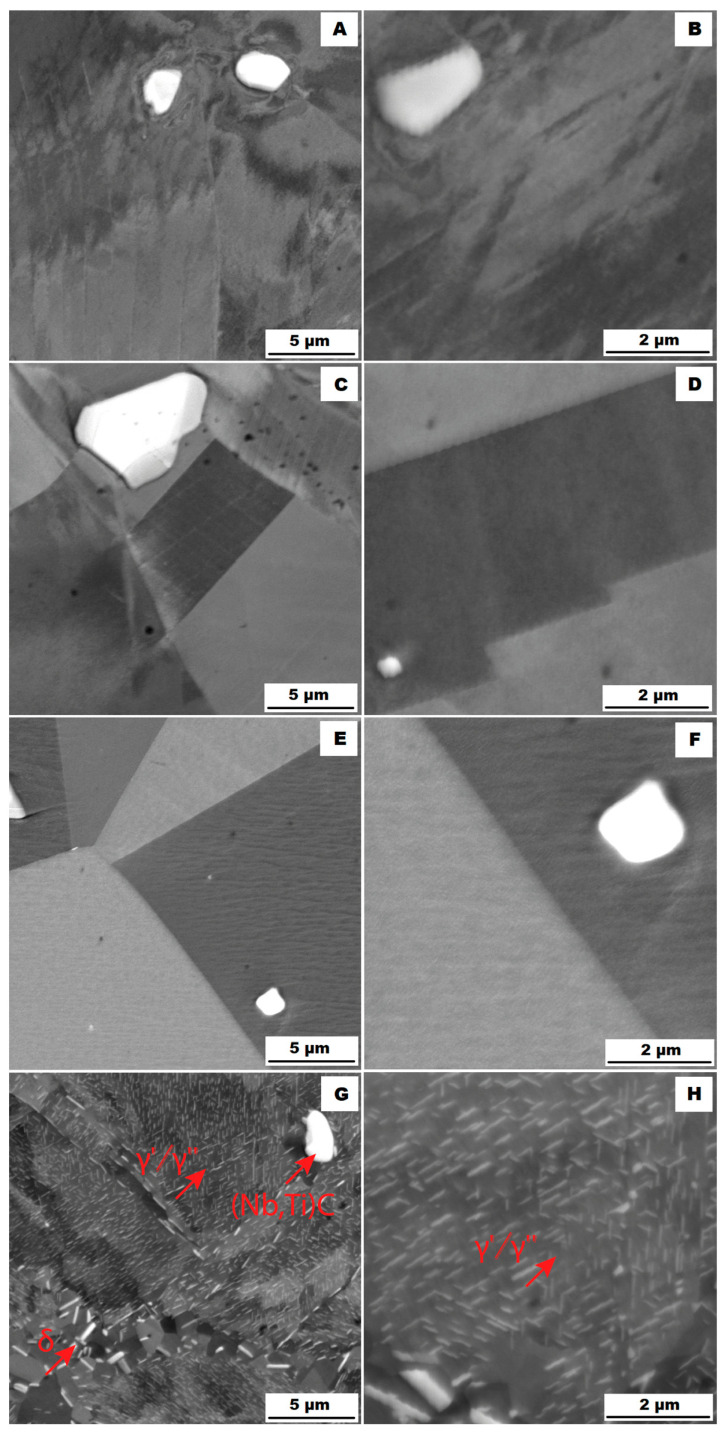
Microstructures of samples ACR HT after the tensile tests (SEM, 10 kV, BSE): (**A**,**B**) tensile test at 600 °C (ACR HT TT600); (**C**,**D**) tensile test at 700 °C (ACR HT TT700); (**E**,**F**) tensile test at 800 °C (ACR HT TT800); (**G**,**H**) tensile test at 900 °C (ACR HT TT900).

**Figure 20:**
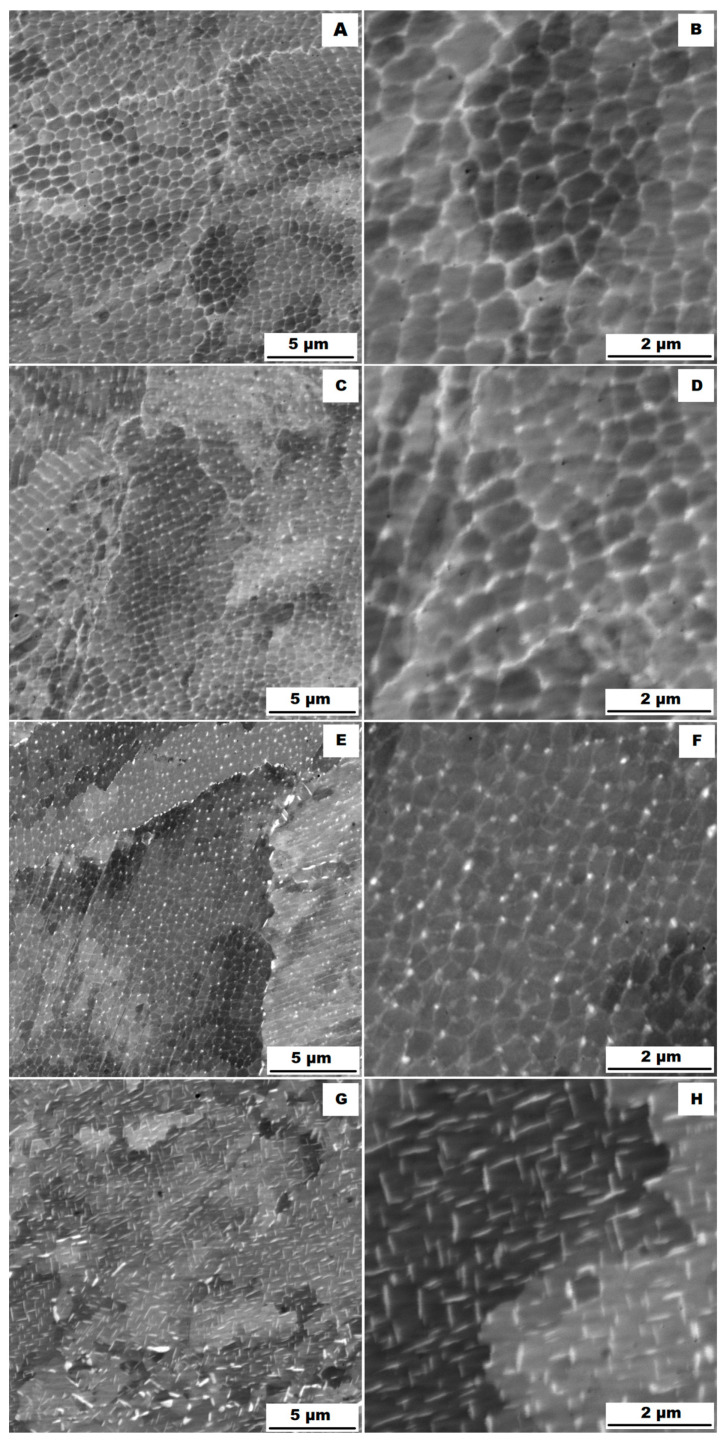
Microstructures of samples SLM after the tensile tests (SEM, 10 kV, BSE): (**A**,**B**) tensile test at 600 °C (SLM TT600); (**C**,**D**) tensile test at 700 °C (SLM TT700); (**E**,**F**) tensile test at 800 °C (SLM TT800); (**G**,**H**) tensile test at 900 °C (SLM TT900).

**Figure 21:**
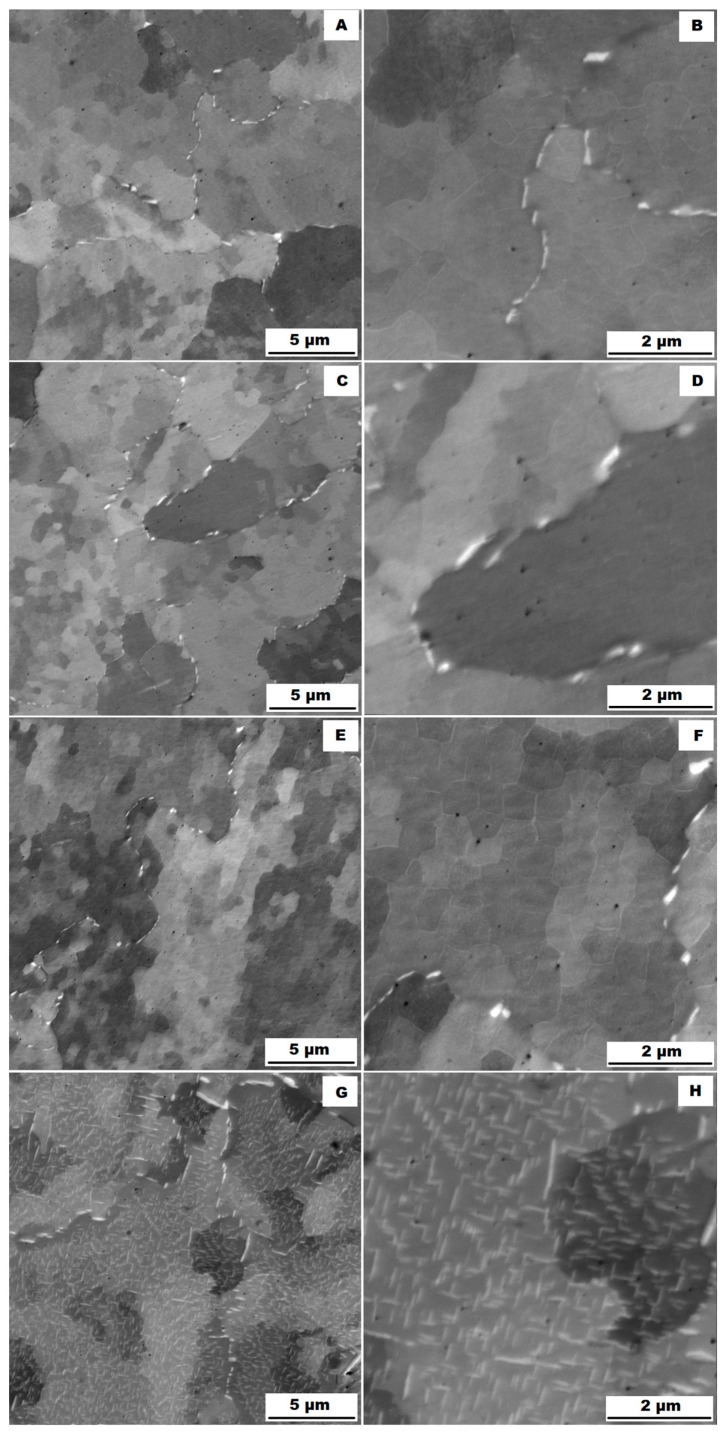
Microstructures of samples SML HT after the tensile tests (SEM, 10 kV, BSE): (**A**,**B**) tensile test at 600 °C (SLM HT TT600); (**C**,**D**) tensile test at 700 °C (SLM HT TT700); (**E**,**F**) tensile test at 800 °C (SLM HT TT800); (**G**,**H**) tensile test at 900 °C (SLM HT TT900).

**Figure 22:**
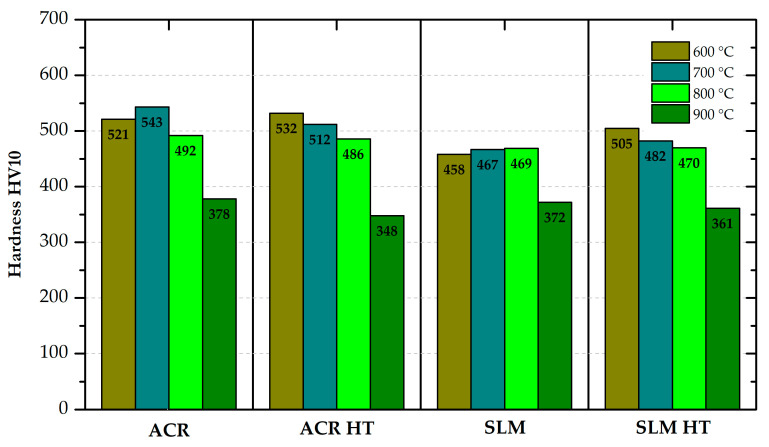
Hardness HV10 after the high-temperature tensile tests (TT).

The study of the alloys’ microstructures after the tensile tests showed that all samples had a different structure after the tensile test at 900 °C (ACR TT900, ACR HT TT900, SLM TT900 and SLM HT TT900 samples) compared with samples tested at 600, 700 and 800 °C. The structures of the individual samples after tensile testing are shown in Figure 18 (ACR sample), Figure 19 (ACR HT sample), Figure 20 (SLM sample) and Figure 21 (SLM HT sample). From these images can be seen that all samples had a structure similar to their relevant initial states after tensile testing at 600, 700 and 800 °C. In contrast, samples of ACR HT TT900 (Figure 19G,H) and SLM HT TT900 (Figure 21G,H) showed a massive increase in the size of the γ′/γ″ phase. Thus, based on the structural analysis, it can be assumed that the strengthening by these γ′/γ″ phases represents the dominant effect up to 800 °C. At higher temperatures, the γ′/γ″ phases become coarser and their hardening effect decreases. In addition, at 900 °C, the solid solution is strongly influenced due to an increase in the size of the γ′/γ″ phase precipitates. The aforementioned coarsened γ′/γ″ phases were also observed in ACR TT900 (Figure 18G,H) and SLM TT900 (Figure 20G,H) after tensile testing at 900 °C. Their precipitation from the solid solution was probably caused by a combination of applied temperature and stress. The statement about the hardening contribution reduction using the solid solution is also supported by the hardness measurements, whose values are summarized in the graph in Figure 22. It can be seen that in samples after tensile tests at 900 °C, where an increase in γ′/γ″ particle size was observed, the hardness decreased in all cases by about 100 HV10 due to the mentioned lower effect of solid solution hardening [32].

The particles of the γ′/γ″ phase start to become coarser above 700 °C and their ability to matrix strengthen and prevent dislocation motion gradually decreases, leading to a sharp decrease in yield stress [33]. However, the γ′/γ″ phase particles are so small up to 800 °C that TEM investigation would be necessary to show them (but their presence in the matrix is confirmed by XRD—Figure 5). The γ′/γ″ particles become coarse enough at 900 °C that they are visible on the SEM figures (Figure 18H, Figure 19H, Figure 20H and Figure 21H). At longer dwell times, γ′/γ″ phase particles gradually dissolve and are substituted by the σ phase [34]. A sudden drop in ductility in the area around 800 °C can also be associated with this phenomenon. Due to the increased temperature, a large portion of γ′/γ″ nanoprecipitates is formed in the alloy, which causes precipitation hardening and a decrease in ductility [33]. The coarsening of the γ′/γ″ phase can also form notches in the matrix that reduce the ductility of the material at this temperature. The further coarsening of the γ′/γ″ phase is observed at 900 °C, but the matrix is already plastic enough at this temperature that the ductility increases significantly regardless of the notch effect caused by the precipitates present, and the alloy begins to reveal superplastic behaviour.

SLM samples revealed half of the ductility value compared with ACR samples, as was also shown in [35]. A similar problem was observed in the determination of notch toughness at room temperature (see Figure 16). While the sample of the rolled material showed impact energy of 249 J·cm^−2^ (ACR sample) and 103 J·cm^−2^ (ACR HT sample), the sample fabricated by the additive technology revealed an impact energy of only 58 J·cm^−2^ (SLM sample) and 35 J·cm^−2^ (SLM HT sample). The lower ductility and notch toughness determined in the SLM samples are probably due to the different failure mechanisms of testing samples. The study of the fracture surfaces showed that while the formed samples (ACR) revealed ductile fracture, samples produced by additive manufacturing (SLM) failed by partly ductile and partly brittle fracture. The brittle fracture results in a reduction of ductility and notch toughness as well [36]. Generally, SLM samples (not only Inconely 718 but also steels, Ti alloys and others) have lower impact energy than cast materials. This is connected with the technology of material creation. In the case of cast samples, melting occurs in the entire volume and then crystallization occurs. For SLM samples, only gradual local melting is performed, so the compactness of the samples is lower. This negative can be eliminated by heat treatment in the case of mechanical properties. In the case of impact energy, improving the values is very problematic [37].

However, the production of parts from Inconel 718 by additive manufacturing is very promising from the point of view of the very low machinability of the material. However, it should be taken into account that the production technology can strongly influence some mechanical properties of the alloy.

For the samples without heat treatment (ACR and SLM), a yield strength anomaly was observed at 700 °C. This increase in yield stress with increasing temperature is also observed for some other alloys (e.g., Fe-Al intermetallics). Many factors contribute to the yield strength anomaly. Among the most important factors is mainly the disintegration of super-dislocations in the highly ordered lattice that exists in the material at lower temperatures, and the transformation of their motion into the motion of separate dislocations. The motion of these separate dislocations is more difficult [38]. Furthermore, the amount of γ′/γ″ phase is a factor in this phenomenon. As the volume of these phases increases, there is additional hardening in the region around 700 °C and thus an increase in the yield stress [39].

## 5. Conclusions

The nickel-iron-based alloy Inconel 718 is a precipitation-hardenable alloy of nickel, chromium, iron and other elements that exhibits high strength at room and elevated temperatures. In this paper, the effect of the used manufacturing method (conventional technology and SLM technology) on the mechanical and structural properties, including the effect of the heat treatment application, was assessed. Based on the performed experiments, the following conclusions can be drawn:

- ACR samples in the as-delivered condition (solution annealing followed by the forming) had the same strength properties as the SLM specimens, but twice the ductility.
- The heat treatment (as described in Section 2) doubled the strength properties of the ACR as well as SLM samples. The strength properties were almost identical for both types of samples over the temperature range RT—900 °C.
- Due to overall lesser compactness, SLM samples after HT showed half of the ductility and notch toughness at the level of about 40%.
- The microstructure of the SLM samples prepared by the parameters in Table 3 showed a very dense, uniformly distributed network of fine δ phase precipitates, while in the matrix of ACR samples, needle-like δ phase precipitates were observed at the grain boundaries and coarse precipitates (Nb, Ti)C were present on the grain surface.
- Heat treatment of the rolled alloy (ACR HT) resulted in significant grain coarsening from 9.18 ± 1.2 to 32.65 ± 20.2 um. In the case of SLM samples, no grain coarsening occurred after HT and the mean grain size remained as 17.51 ± 10.9 um.
- The δ phase precipitates were dissolved into the matrix during heat treatment and probably replaced by the coherent γ′/γ″ phase precipitates. Coarse secondary (Nb, Ti)C particles remained presented in the structure. In the case of material produced by additive manufacturing (SLM HT), heat treatment caused a redistribution of secondary particles. The dense network of fine precipitates was dissolved into the matrix and coherent precipitates of γ’ and γ″ phases were formed.

## Figures and Tables

**Table 1:** Chemical composition—Inconel 718 (rolled samples).

Wt. %	Ni	Cr	Fe	C	Mn	Si	Mo	Nb
	53.12	19.33	Bal.	0.036	0.091	0.0012	3.168	5.38
Wt. %	Al	Ti	Co	Cu	W	P	V	B
	0.542	0.926	0.187	0.048	0.032	0.0012	0.024	0.0041

**Table 2:** Chemical composition—the powder Inconel 718 (3D).

Wt. %	Ni	Cr	Fe	C	Mn	Si	Mo	Nb
	52.04	18.76	Bal.	0.03	0.03	0.04	3.07	5.01
Wt. %	Al	Ti	Co	Cu	W	P	V	B
	0.53	1.0	0.1	0.02	-	0.004	-	-

**Table 3:** Process parameters used at sample fabrication by additive technology.

**Parameter**	**Thickness of Layer [µm]**	**Power of Laser [W]**	**Scanning Rate [mm/s]**	**Hatch Spacing [µm]**	**Shielding Atmosphere**
**Values**	30	200	900	120	Ar

**Table 4:** Determined grain size in dependence on tested material and application of heat treatment.

Samples without HT	Grain Size [µm]	Samples with HT	Grain Size [µm]
ACR	9.18 ± 1.20	ACR HT	32.65 ± 20.28
SLM	17.51 ± 10.99	SLM HT	17.26 ± 10.90

**Table 5:** Basic material properties of the tested materials at room temperature.

Samples	Proof Yield Strength	Ultimate Tensile Strength	Uniform Ductility	Total Ductility
	*R*_p0.2_ (MPa)	*R*_m_ (MPa)	*A*_g_ (%)	*A*_40mm_ (%)
ACR	540	958	35.2	39.3
ACR HT	1147	1370	16.1	24.1
SLM	581	874	18.3	21.3
SLM HT	1190	1299	10.3	14.8

## Data Availability

The raw/processed data required to reproduce these findings cannot be shared at this time due to legal or ethical reasons.
